# Effects of *CYP2E1* Polymorphism and Dietary Supplementation With Jerusalem Artichoke on Skatole and Indole Levels in the Back Fat and Feces of Boars, and Liver mRNA Expression

**DOI:** 10.1002/fsn3.72272

**Published:** 2026-09-03

**Authors:** Kateřina Zadinová, Antonín Stratil, Jaroslav Čítek, Monika Okrouhlá, Jan Calta, Ivan Bahelka, Kateřina Urbancová, Michal Šprysl, Roman Stupka

**Affiliations:** ^1^ Department of Animal Science, Faculty of Agrobiology, Food and Natural Resources Czech University of Life Sciences Prague Prague Czech Republic; ^2^ Laboratory of Molecular Ecology Institute of Animal Physiology and Genetics, Academy of Sciences of the Czech Republic Liběchov Czech Republic; ^3^ Department of Genetics and Breeding, Faculty of Agrobiology, Food and Natural Resources Czech University of Life Sciences Prague Prague Czech Republic

**Keywords:** boars, *CYP2E1*, Jerusalem artichoke, mRNA expression, nutrition, skatole

## Abstract

The objective of this study was to evaluate the effects of adding Jerusalem artichoke to the diet of fattening boars and the effects of polymorphisms in the *CYP2E1* gene on skatole levels in back fat and feces and on *CYP2E1* mRNA expression in pig liver. A total of 25 crossbreed boars (LW_S_ × Pn) × (L_D_ × LW_D_) were used in this study. All boars were genotyped for c.1423G > A. The pigs were divided into two different dietary treatment groups with 0% (C—control; *n* = 12) and 8.2% (E—experimental; *n* = 13) dried Jerusalem artichoke tubers, which were fed 14 days before slaughter. The results suggest that feeding Jerusalem artichoke decreased (*p* = 0.0236) the level of skatole in adipose tissue. In feces, a decrease in the skatole level was observed, but there was no statistically significant difference between the control and experimental groups. The influence of polymorphism on skatole levels in adipose tissue and feces were lowest for the *AA* genotype and highest for the *GG* genotype. Differences between genotypes for skatole levels in feces were statistically significant (*p* = 0.0242). Differences between genotypes in adipose tissue on skatole levels showed the same trend in favor of the *A* allele. However, these differences were not statistically significant. This study also confirmed the significant effect of Jerusalem artichoke on the liver *CYP2E1* mRNA expression. The *CYP2E1* gene expression level was significantly higher in the experimental group (*p* = 0.0086). No significant differences in mRNA expression between different genotypes were detected.

## Graphical abstract

This study investigated the combined effects of *CYP2E1* gene polymorphism (SNP NM_214421.1: c.1423G > A) and short‐term dietary supplementation with Jerusalem artichoke ( *Helianthus tuberosus* , 8.2% tuber meal) on skatole and indole concentrations in back fat and feces of 25 crossbreed boars, and on hepatic *CYP2E1* mRNA expression. Animals were assigned to a control group (C, *n* = 12, standard feed) or an experimental group (E, *n* = 13, supplemented feed for 14 days before slaughter) and were genotyped as *AA* (*n* = 5), *AG* (*n* = 12), or *GG* (*n* = 8). Jerusalem artichoke supplementation significantly reduced skatole concentrations in back fat (*p* = 0.024) and increased *CYP2E1* mRNA expression in liver (*p* = 0.009). The *GG* genotype was associated with significantly higher skatole concentrations in feces compared with the *AG* and *AA* genotypes (*p* = 0.024). These findings indicate that short‐term inclusion of Jerusalem artichoke in the diet modulates boar taint compounds through *CYP2E1*‐mediated hepatic metabolism, and that *CYP2E1* genotype is associated with differences in intestinal skatole production. The results support dietary manipulation as a practical, nonsurgical strategy to reduce boar taint in entire male pigs.

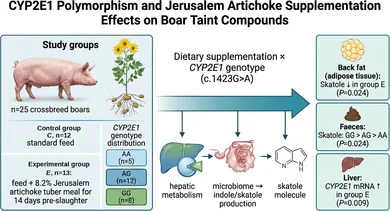

**Abbreviations**
ACTB: actin beta
ADG: average daily gain
B2M: beta‐2 microglobulin
C: control
CYP2E1: cytochrome P450 family 2 subfamily E member 1
DNA: deoxyribonucleic acid
E: experimental
FCR: feed conversion ratio
FI: daily feed intake
GIT: gastrointestinal tract
HPLC/FLD: high‐performance liquid chromatography with fluorescence detection
HPRT1: hypoxanthine phosphoribosyltransferase 1
K_2_EDTA: dipotassium‐ethylenediaminetetraacetic acid
L_D_
: Landrace dam line
LW: live weight
LW_D_
: Large White dam line
LW_S_
: Large White sire line
MDGC/MS: multidimensional gas chromatography–mass spectrometry
mRNA: messenger ribonucleic acid
PCR: polymerase chain reaction
Pn: Pietrain
qPCR: quantitative polymerase chain reaction

## 1 Introduction

Boars have better feed conversion, faster growth rates, and leaner meat than surgically castrated male pigs (Landferdini et al. 2013). However, in most countries, male pigs are still castrated very soon after birth to avoid the production of boar taint compounds (Bonneau and Weiler 2019; Morales et al. 2017). These unpleasant odor compounds constitute one of the main problems in the pork industry production of entire male pigs (Wesoly and Weiler 2012). Boar taint is usually described by consumers as a strong, fecal and urine‐like odor (Rowe et al. 2014). The main components responsible for boar taint are 5α‐androst‐16‐en‐3‐one (androstenone), 3‐methylindole (skatole), and 1H‐indole (indole) (Drag et al. 2018). Both androstenone and skatole can be affected by genetic and environmental factors, but skatole is more sensitive to housing conditions and nutritional management (Brinke et al. 2020). The production of boar taint compounds increases during sexual maturity because of reduced metabolism in the liver (Zamaratskaia et al. 2004; Vhile et al. 2012).

Surgical castration represents a problem from an ethical point of view and also endangers the welfare of pigs in general, as this type of young boar castration, which is carried out a few days after birth, is painful, frightening, and can lead to a decline in health (de Campos et al. 2015; Lundström and Zamaratskaia 2006). An alternative could be fattening uncastrated male pigs with feed supplements that block the formation of boar taint compounds (Urbanová et al. 2016; Aluwé et al. 2017). In addition to feeding strategies and the use of feed additives, genetic selection toward lower boar taint incidence could help pig farmers shift toward raising boars (Van Den Broeke et al. 2015). A recent review by Squires and Bone (2025) confirmed that an integrated approach combining genetic selection, immunocastration, and nutritional strategies—including prebiotic supplementation—represents the most promising pathway to eliminating surgical castration while maintaining acceptable pork quality.

Skatole has a characteristic unpleasant, fecal odor that is formed by L‐tryptophan degradation in the gastrointestinal tract (GIT) of boars. This compound is produced in both sexes of pigs, but increased accumulation is observed in some boars. The production of this compound in the hindgut can be affected by the diet used (Bee et al. 2020). Earlier studies reported that feeding pigs diets supplemented with carbohydrates, for example, Jerusalem artichoke fermented in the hindgut, contributes to reducing skatole levels in the hindgut and subsequently to decreasing its accumulation in adipose tissue (Urbanová et al. 2016). “Inulin‐type fructans” are carbohydrates that include all *β* (2 ← 1) linear fructans. These bacteria are resistant to hydrolysis by enzymes in the small intestine but are easily fermented by certain bacteria in the hindgut (primarily bifidobacteria) (Flickinger et al. 2003). Thus, changing the feeding strategy results in the reduced production of skatole and indole in the GIT of boars (Kjos et al. 2010; Salmon and Edwards 2015).

Skatole and indole produced by the GIT are further metabolized in the liver. The initial step of skatole metabolism in the liver involves mainly the enzyme cytochrome P450 family 2 subfamily E member 1 (CYP2E1), which is encoded by the *CYP2E1* gene (Zamaratskaia and Squires 2009). Earlier studies reported a negative correlation between *CYP2E1* mRNA expression or enzyme activity and the concentration of skatole in adipose tissue (Bilič‐Šobot et al. 2016; Zadinová et al. 2021). Whittington et al. (2004) described low hepatic *CYP2E1* gene expression in crossbred Meishan × Large White pigs. The activities of enzymes or other proteins can be affected by mutations or variations in coding regions of genes. The association of the *CYP2E1* SNP c.1423G > A (NM_214421.1) with the skatole level in backfat was described by Mörlein et al. (2012) and Zadinová et al. (2017). The most favorable genotype (for low skatole and indole contents) was c.1423 *AA*, represented as allele *A*. Archer et al. (2024) identified single‐nucleotide polymorphisms in the promoter region of the *CYP2E1* gene that were associated with *CYP2E1* mRNA expression. These findings suggest that factors that regulate the translation of *CYP2E1* mRNA may also be important in affecting skatole metabolism. Before selecting against skatole, it is crucial to evaluate the potential effects of such selection on commercial traits measured in crossbreds (Faggion et al. 2023).

Building upon our foundational research demonstrating the impact of *CYP2E1* polymorphisms on adipose tissue skatole levels (Zadinová et al. 2017) and Jerusalem artichoke supplementation's dual effect on reducing skatole deposition while upregulating hepatic *CYP2E1* mRNA expression (Zadinová et al. 2021), this study extends the investigative scope through two novel dimensions. First, we systematically evaluate the functional consequences of the *CYP2E1* SNP c.1423G > A variant on transcriptional regulation using qPCR quantification. Second, we introduce fecal skatole monitoring as a potential proxy for adipose tissue accumulation—an approach never previously examined in the context of dietary interventions targeting boar taint mitigation.

The objective of the present study was to investigate the combined effects of dietary Jerusalem artichoke supplementation and the SNP c.1423G > A in the *CYP2E1* gene on skatole and indole levels in back fat and feces as well as on *CYP2E1* mRNA expression in pig liver. Based on previous findings, the hypothesis was formulated that both Jerusalem artichoke supplementation and the presence of the c.1423G > A SNP in the *CYP2E1* gene would significantly influence hepatic *CYP2E1* mRNA expression and modulate skatole and indole levels in back fat and feces.

## 2 Materials and Methods

### 2.1 Animals and Study Design

Twenty‐five uncastrated male pigs of a commercial crossbred population (Large White sire line × Pietrain) × (Landrace dam line × Large White dam line), denoted (LW_S_ × Pn) × (L_D_ × LW_D_) from 11 litters, were used in this study. On the 3rd day after birth, the piglets were genotyped for c.1423G > A in *CYP2E1*. The pigs were divided into two different dietary treatment groups: group with 0% (C—control; *n* = 12) and group with 8.2% (E—experimental; *n* = 13) supplementation with dried Jerusalem artichoke ( *Helianthus tuberosus* ), which was fed at 140 days of age, 14 days before slaughter. The distribution of *CYP2E1* genotypes (c.1423G > A) in both groups was equal (control group: *AA n* = 2, *AG n* = 6, *GG n* = 4; experimental group: *AA n* = 3, *AG n* = 6, *GG n* = 4).

The pigs were identified by means of electronic ear chips. The pigs were maintained at a testing station (three pigs per pen) under the standard husbandry conditions described in our previous publication (Dvořáková et al. 2012). The animals were fed ad libitum with basal complete feed mixtures. The composition was continuously adjusted to the age and weight of the pigs and is described in Table 1. The experimental group was fed dried Jerusalem artichoke for 14 days before slaughter. The pigs were slaughtered at 154 days of age, and the average live weight was ±108.6 kg.

**TABLE 1:** Ingredients and chemical composition of the boars' diets.

Indicator	Diets		
	C + E	C + E	E
	P2	P3	P3 + 8.2% JA
Average live weight (kg)	45.7–66	66–108.6	96.5–108.6
Age of pigs (days)	89–110	111–153	140–153
Components (g/kg)			
Barley	503	390	490
Wheat	310	490	388
Soybean meal	150	90	10
Premix of vitamins and minerals^a^	30	30	30
Monocalcium phosphate	7	—	—
Jerusalem artichoke (JA)	—	—	82
Chemical composition			
Dry matter (%)	90.95	87.3	87.3
Crude protein (%)	16.4	14.8	14.8
Crude fat (%)	1.06	1.06	1.06
Crude fiber (%)	3.7	3.63	3.63
Starch (%)	45.4	50.8	50.8
ME3 (MJ/kg)	13.2	12.8	12.6
Lysine/ME	0.73	0.60	0.60
Amino acids (g/kg)			
Lysine	9.64	11.2	11.2
Methionine	2.95	2.81	2.81
Threonine	6.24	5.39	5.39
Tryptophan	2.06	1.74	1.74

Abbreviations: C, control dietary treatment group; E, experimental dietary treatment group; P2, complete feed mixture for the 1st phase of fattening; P3, complete feed mixture for the 2nd phase of fattening.

^a^ Premix of micro‐ and macrominerals, essential amino acids, and vitamins. One kilogram of premix provided: retinol, 400,000 IU; Cholecalciferol, 66,000 IU; α‐tocopherol, 3600 mg; menadione, 100 mg; thiamine, 60 mg; riboflavin, 150 mg; niacin, 800 mg; Ca pantothenate, 375 mg; vitamin B6, 100 mg; vitamin B12, 1 mg; choline Cl, 15,000 mg; folic acid, 15 mg; Fe, 3500 mg as FeSO_4_·H_2_O; Zn, 3600 mg as ZnO; Mn, 3100 mg as MnO; Cu, 330 mg as CuSO_4_·5H_2_O; I, 175 mg as Ca(IO_3_)_2_; Co, 15 mg as 2CoCO_3_·3Co(OH)_2_·H_2_O; Se, 13 mg as Na_2_SeO_3_; 6‐phytase (EC 3.1.3.26), 25,000 FTU; Ca, 220 g; P, 20 g; Na, 50 g; Mg, 10 g; lysine, 85 g; methionine, 15 g; threonine, 15 g.

The dried Jerusalem artichoke ( *Helianthus tuberosus* L.) tubers were purchased from FeedNatursro (Czech Republic). As a commercially produced agricultural product, species identity is documented by the supplier's product declaration; no herbarium voucher specimen was deposited.

### 2.2 Sample Collection

Blood samples for DNA isolation and genotyping from the experimental crossbred pigs were collected into tubes with K_2_EDTA 3 days after birth and stored at 20°C.

Samples for skatole and indole analyses were collected from pig back fat from the neck region between the first and third cervical vertebrae 1 day after the slaughter of the boars. The samples were vacuum packed and frozen at −80°C until chemical analysis.

Liver tissue samples (150 mg) were collected from the left lateral lobe of the liver at slaughter within 30 min post mortem. These samples were submerged in RNAlater Stabilization Solution (Thermo Fisher Scientific, Waltham, Massachusetts, USA) and stored at −20°C until analysis.

Feces samples (ca. 5 g of feces) from all boars were collected during their defecation in three periods directly from the rectum. The first samples were collected 15 days before slaughter (1 day before application of the Jerusalem artichoke feeding); the second samples were collected 7 days later (during the Jerusalem artichoke feeding), and the last samples were collected 1 day before slaughter (after the feeding was terminated). The samples were collected into 20 mL polypropylene tubes containing anhydrous silica gel on the bottom, which passively absorbed moisture from the fecal material through vapor‐phase equilibration; the residual moisture content was between 3% and 5%, verified gravimetrically. This sample stabilization procedure was adapted from validated analytical methods developed in our laboratory (Doležal et al. 2020, 2022). The samples were stored at 5°C and analyzed by MDGC/MS (GC‐2010, Shimadzu, Kyoto, Japan) no later than 12 h after collection, using 1,2‐dimethylindole as an internal standard (linearity 1–70 μg/g; recovery 91%–98%; LOQ: 1.6 μg/g for indole, 2.0 μg/g for skatole). Concentrations are expressed per gram of dried fecal sample (3%–5% residual moisture); no correction to fresh weight basis was applied, as all samples were processed under identical drying conditions.

### 2.3 Isolation of DNA, PCR, and Genotyping

Genomic DNA was isolated from blood using a Genomic DNA Mini Kit (Blood/Cultured Cell) (Geneaid Biotech, New Taipei City, Taiwan). The DNA samples were stored at −20°C until use. The PCR fragments of *CYP2E1* were amplified by polymerase chain reaction (PCR) using the primers *CYP2E1‐AB* (*CYP2E1‐A*: 5′‐GCTTAGGGTGATGGTTTACACA‐3′ and *CYP2E1‐B*: 5′‐GGAACCCAACACAGACTCAA‐3′). Polymerase chain reaction was performed in 25 μL reaction volumes with 100 ng of genomic DNA, standard reaction buffer, and 1.5 mM MgCl_2_, 200 μM each dNTP, 10 pmol of each primer, 2% DMSO, and 1 U of LA polymerase (Top‐Bio, Prague, Czech Republic). The PCR profile was 2 min at 95°C, followed by 30 cycles of 30 s at 95°C, 30 s at 55°C, and 45 s at 68°C, with a final extension of 5 min at 68°C. The PCR fragment (524 bp) was visualized on a 1.5% agarose gel. Pigs were genotyped for the SNP NM_214421.1:c.1423G > A using restriction fragment length polymorphism analysis of PCR‐amplified fragments (PCR–RFLP) with the enzymes *Bsp*68I, *Hpy*188III, and *Bsr*DI (c.1423G > A.) according to Zadinová et al. (2017). The *CYP2E1* gene contains two adjacent SNPs in exon 9 at consecutive nucleotide positions: c.1422C > T and c.1423A > G. Due to this immediate proximity, three restriction enzymes (B*sp*68I, H*py*188III, and B*sr*DI) were used in combination to unambiguously distinguish all possible genotype combinations. The c.1423A > G genotype was determined based on the digestion patterns of H*py*188III and B*sr*DI. The detailed protocol has been described previously (Zadinová et al. 2017).

### 2.4 Isolation of RNA, cDNA Synthesis and Quantitative Polymerase Chain Reaction (qPCR)

Total RNA was isolated using an Aurum Total RNA Fatty and Fibrous Tissue Kit (Bio‐Rad Laboratories Inc., Hercules, California, USA). The quantity and purity of the RNA samples were analyzed with a Nanodrop ND‐1000 spectrophotometer (Thermo Fisher Scientific, Waltham, Massachusetts, USA). The RNA concentration ranged from 589 ng/μL to 1408 ng/μL. cDNA was synthesized using an Improm‐II cDNA synthesis kit (Promega, Madison, WI, USA). qPCR was performed according to Zadinová et al. (2021) on a CFX96 Touch Real‐Time PCR Detection System (Bio‐Rad Laboratories Inc.) in 10 μL reaction volumes using 5 μL of KAPA SYBR FAST qPCR master mix (KAPA Biosystems, MA, USA). All qPCRs were performed at least two times. The reference genes (*ACTB*, *HPRT1*, and *B2M*) were selected based on previous research. PCR primers for reference genes and primers independent of the specific transcript variant for the exon‐spanning analysis of *CYP2E1* were obtained from the publications of Erkens et al. (2006) and Zadinová et al. (2021).

### 2.5 Determination of Skatole and Indole Levels in Fat and Feces Samples

Skatole and indole levels in back fat were determined by high‐performance liquid chromatography with fluorescence detection (HPLC/FLD) (LC‐2000Plus HPLC system; Jasco, Tokyo, Japan) using the method of (Hansen‐Møller 1994), which was modified from (Okrouhlá et al. 2016). The determination of boar taint compounds using a Kinetex C18 100A (5 μm, 50 × 4.60 mm ID) column was carried out at 40°C. The mobile phase parameters were as follows: A—potassium phosphate buffer (10 mM) and B—methanol. The gradient profile was as follows: 0–0.2 min, 90% A; 0.2–6.0 min, 90%–55% A; and 6.0–7.0 min, 55%–0% A. The column flow rate was 1.2 mL/min, with an injection volume of 30 μL. Fluorescence detection was performed with excitation at 285 nm and emission at 340 nm. A standard calibration curve was used for determination of the concentrations of skatole and indole.

Skatole and indole levels in feces samples were measured by multidimensional gas chromatography–mass spectrometry (MDGC/MS). The samples were prepared according to the following protocol. For the analysis, 5 ± 0.1 g samples of feces from individual boars were weighed into clean glass vials. Ultrapure water (10 mL) was added, and the vials were shaken for 20 min on a shaker (Shaker GFL 3006, Germany). All the resulting fecal slurries were centrifuged at 1900× *g* for 5 min (Hettich Universal 320R, Verkon Ltd., *town*, Czech Republic). The aqueous supernatants (4 mL) were pipetted into new clean vials and recentrifuged at 1900× *g* for 5 min to obtain Solution A. A 2 mL aliquot of the Solution A supernatant was then pipetted into new clean vials (5 mL). Next, 450 μL of chloroform and 50 μL of internal standard (IS) (1,2‐dimethylindole, 100 μg/mL) were added to each vial, and the mixtures were shaken for 1 h. The final solutions were then centrifuged at 1900× *g* for 5 min. For the analyses, 200 μL of each chloroform phase was pipetted into a glass insert vial containing several anhydrous sodium sulfate crystals. The sample preparations were carried out at a constant laboratory temperature (22°C) and under standard atmospheric pressure. The methodology was based on previous studies (Doležal et al. 2020, 2022).

### 2.6 Growth Performance

All boars were weighed individually at weekly intervals, and feed intake was monitored daily. The live weight (LW), daily feed intake (FI), feed conversion ratio (FCR), and average daily gain (ADG) were calculated based on the obtained values. After slaughtering, the carcass weight (CW) was measured. For the qualitative parameters, the intramuscular fat content (IMF) was measured.

### 2.7 Statistical Analyses

The response variables of fat‐indole and fat‐skatole were analyzed in SAS 9.4 (Statistical Analysis System, Inst. Version 9.4, 2012, SAS Institute, Cary, NC, USA) using a fixed‐effects model (PROC GLM) with the nutritional category and the *CYP2E1* genotype as predictors.

$$Y_{\mathrm{ij}}=\mu +{\text{Diet}}_{i}+{\text{Genotype}}_{j}+\varepsilon_{\mathrm{ij}}$$

where *Y*_*ij*_ is the observed value, *μ* is the overall mean, Diet_*i*_ is the fixed effect of dietary treatment (*i* = Control, Jerusalem artichoke), Genotype_*j*_ is the fixed effect of *CYP2E1* genotype (*j* = *AA*, *AG*, *GG*), and *ε*_*ij*_ is the random residual error.

Other response variables, which were measured repeatedly, were analyzed using a mixed‐effects model (PROC MIXED) that included nutrition, genotype, and order of measurement as fixed effects and animal ID as a random effect.

$$\begin{array}{c} Y_{\text{ijkl}}=\mu +{\text{Diet}}_{i}+{\text{Genotype}}_{j}+{\text{Measurement}}_{k}\\ \;+{\text{Animal}}_{l}\;\left({\text{Diet}}_{i}\times {\text{Genotype}}_{j}\right)+\varepsilon_{\text{ijkl}} \end{array}$$

where *Y*_*ijkl*_ is the observed value, *μ* is the overall mean, Diet_*i*_, Genotype_*j*_, and Measurement_*k*_ are fixed effects, Animal_*l*_ is a random effect nested within Diet × Genotype, and *ε*_*ijkl*_ is the random residual error.

Furthermore, we estimated a partial correlation coefficient between the variable's fat‐skatole and feces‐skatole‐3 while controlling for the effects of nutrition and SNP (PROC GLM with MANOVA and printE options).

The normalized relative *CYP2E1* expression values (NRD‐*CYP2E1*), calculated using the geometric mean of three reference genes (*ACTB*, *B2M*, and *HPRT1*), were log_10_‐transformed prior to statistical analysis, as qPCR expression ratios are multiplicative in nature and typically follow a log‐normal distribution (Livak and Schmittgen 2001; Pfaffl 2001). Normality of the log‐transformed data was assessed using the Shapiro–Wilk test; homogeneity of variance across genotype groups was evaluated using Levene's test. One animal (No. 4, *AA* genotype) was identified as a statistical outlier by the Grubbs test (G = 3.70, G_crit = 2.82, *p* < 0.05) but was retained in all analyses, as its exclusion would have further reduced the smallest genotype group (*AA*, *n* = 5) and introduced bias into the between‐genotype comparison. The log‐transformed data were analyzed by one‐way analysis of variance (ANOVA). The results are presented as the least squares means (LSMs) with standard errors (SEs). Differences between LSMs were determined by Duncan's test. The level of significance was set at a *p* < 0.05.

## 3 Results

### 3.1 Effects of Jerusalem Artichoke Feeding on the Levels of Skatole and Indole in Back Fat and Feces

Feeding Jerusalem artichoke decreased the level of skatole in adipose tissue (Table 2). A significant difference (*p* = 0.0236) in back fat content was detected between the control and experimental groups after slaughter. The level of indole in adipose tissue was the same in both the control and experimental groups.

**TABLE 2:** Levels of skatole and indole in feces during the Jerusalem artichoke feeding period and levels of skatole and indole in back fat after slaughter.

Traits	Diet group		
	Control (*n* = 12) LSM ± SE	Experimental (*n* = 13) LSM ± SE	*P*
Skatole, back fat [μg/g]	0.043^a^ ± 0.01	0.026^b^ ± 0.01	0.0236
Indole, back fat [μg/g]	0.087 ± 0.01	0.085 ± 0.004	NS
Skatole, feces [μg/g]	8.475 ± 0.71	7.261 ± 0.66	NS
Indole, feces [μg/g]	9.485 ± 0.84	10.114 ± 0.77	NS

*Note:* Values with different superscripts within one row are significantly different at a and b, *p* < 0.05.

Abbreviations: LSM, least squares means (μg/g); NS, not significant; SE, standard error.

Both the skatole and indole levels measured in feces were several times higher than the levels measured in adipose tissue. No statistically significant differences in skatole or indole levels were detected between the control and experimental groups. However, there was a marked decrease in skatole levels in the experimental group fed Jerusalem artichoke.

### 3.2 Effect of the *CYP2E1* SNP on Skatole and Indole Levels in Back Fat and Feces

To study the effect of the SNP c.1423G > A polymorphism on skatole and indole levels in back fat and feces, the pigs from both the experimental and control groups were joined. The difference in skatole contents between back fat and feces was associated with SNP polymorphisms (see Table 3). For the observed variables, the *AA* genotype presented the lowest levels of skatole. Skatole levels in adipose tissue and feces were lowest for the *AA* genotype and highest for the *GG* genotype. Differences between genotypes for skatole levels in feces were statistically significant (*p* = 0.0242). Differences between genotypes in adipose tissue skatole levels showed the same trend in favor of the *A* allele. However, these differences were not statistically significant.

**TABLE 3:** Effects of *CYP2E1* (c.1423G > A) genotypes on the levels of skatole and indole in back fat after slaughter and in feces during Jerusalem artichoke feeding period.

Traits	*CYP2E1* c.1423G > A			
	*AA* (*n* = 5) LSM ± SE	*AG* (*n* = 12) LSM ± SE	*GG* (*n* = 8) LSM ± SE	*P*
Skatole back fat [μg/g]	0.027 ± 0.01	0.034 ± 0.01	0.043 ± 0.01	NS
Indole back fat [μg/g]	0.084 ± 0.01	0.087 ± 0.004	0.088 ± 0.01	NS
Skatole, feces [μg/g]	6.341^a^ ± 1.05	7.329^a^ ± 0.68	9.933^b^ ± 0.83	0.0242
Indole, feces [μg/g]	8.532 ± 1.24	11.532 ± 0.8	9.328 ± 0.98	NS

*Note:* Values with different superscripts within one row are significantly different at a and b, *p* < 0.05.

Abbreviations: LSM, least squares means (μg/g); NS, not significant; SE, standard error.

According to the results, the levels of indole in both fat and feces were not significantly influenced by the genotype of the *CYP2E1* gene.

A partial correlation (*r* = 0.368) between the level of skatole in adipose tissue and the level of skatole in feces after controlling for diet and SNP seemed to be insignificant (*p* = 0.092).

### 3.3 *CYP2E1* mRNA Expression in Boar Livers

**FIGURE 1:**
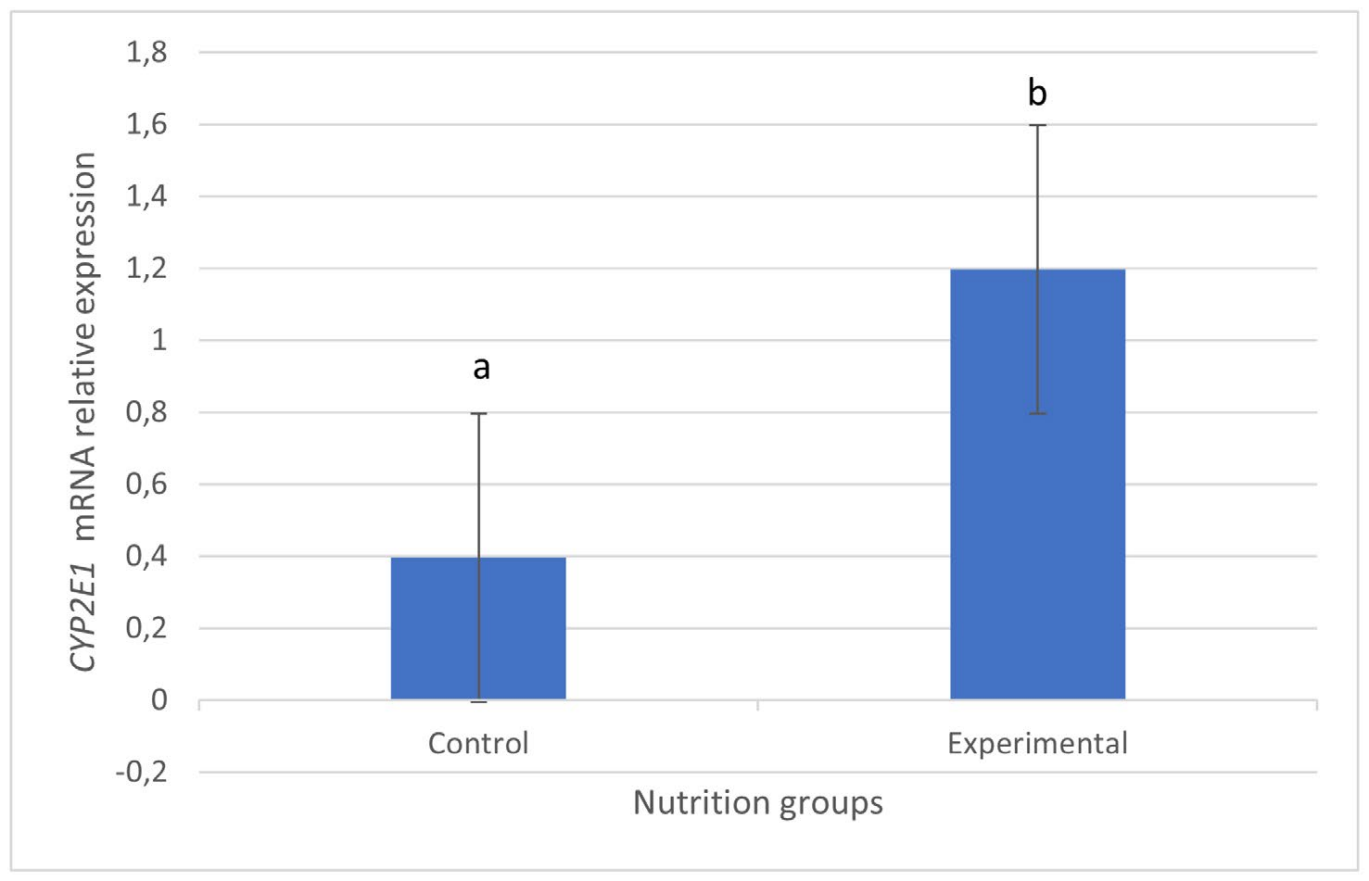
Effects of Jerusalem artichoke feeding on changes in *CYP2E1* mRNA expression in boar liver. Least squares means (± SD) of the log_10_‐transformed relative mRNA expression of *CYP2E1* in liver from entire male pigs. Boars were divided into two dietary treatment groups: A control group without Jerusalem artichoke, and an experimental group fed a diet containing 8.2% of dried Jerusalem artichoke in feed rations for 14 days before slaughter. Error bars represent standard deviations (SD). Bars with different letters are significantly different at: a, b—*p* < 0.05.

**FIGURE 2:**
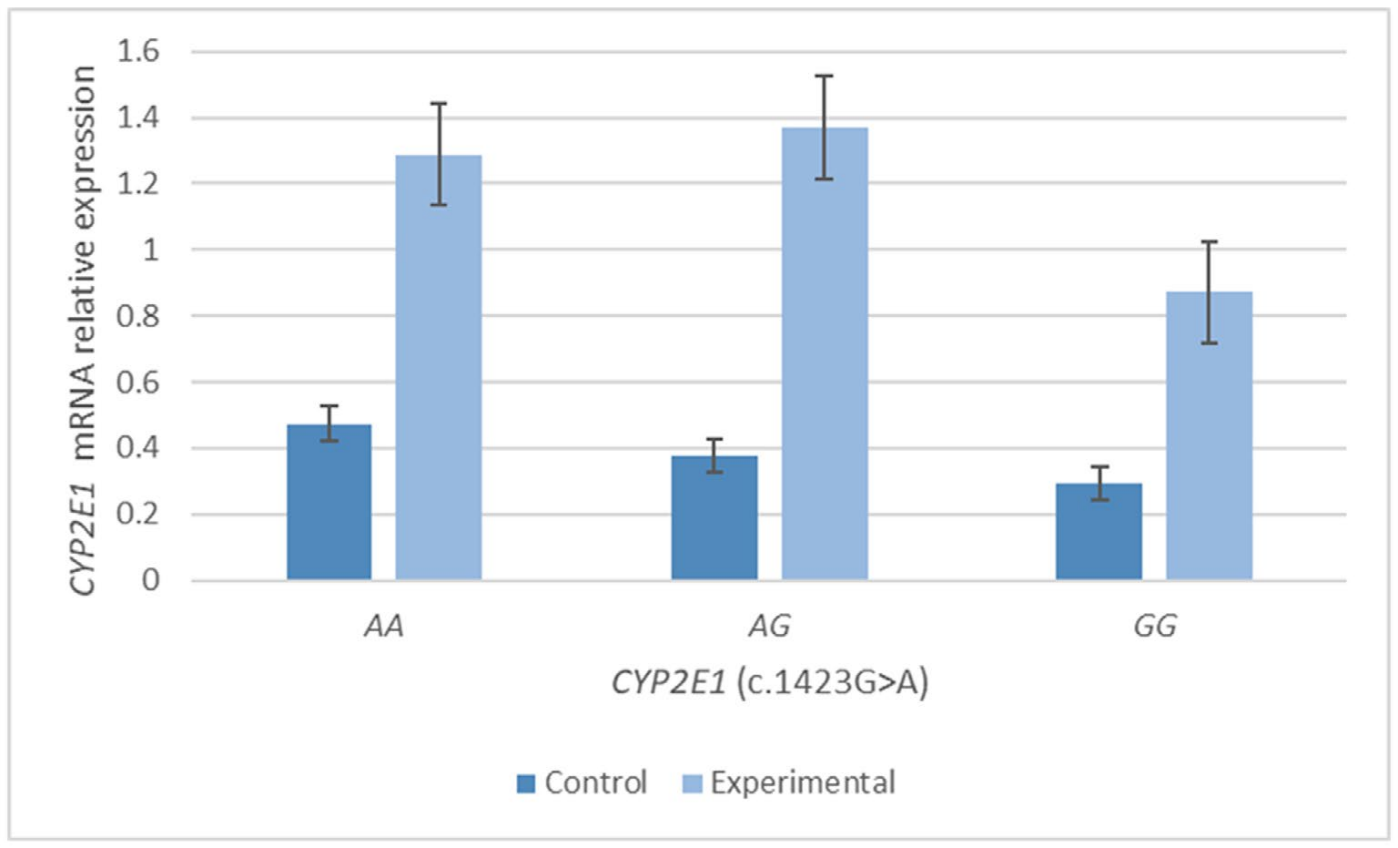
Effect of *CYP2E1* polymorphism on *CYP2E1* mRNA expression in boar liver. Least squares means (± SD) of the log_10_‐transformed relative mRNA expression of *CYP2E*1 in liver from entire male pigs. Boars were divided according to genotype *CYP2E1* c.1423G > A. Error bars represent standard deviations (SD) of the log_10_‐transformed *CYP2E1* mRNA relative expression values (NRD‐*CYP2E1*). The larger SD in the *AG* experimental group reflects the biological heterogeneity inherent to heterozygous individuals carrying two functionally distinct allelic variants of *CYP2E1*.

The specificity of the *CYP2E1* qPCR amplicon was confirmed in a previous publication (Zadinová et al. 2021). The level of *CYP2E1* mRNA expression was significantly greater in the experimental group (with the addition of Jerusalem artichoke) than in the control group (*p* = 0.0086) (Figure 1). Comparison of expression levels of different genotypes, both in the control and experimental groups, did not show significant differences between the genotypes (Figure 2).

When the effect of the polymorphism in the *CYP2E1* gene was evaluated, the lowest levels were observed in boars with the *GG* genotype, compared with the *AA* and *AG* genotypes.

### 3.4 Growth Performance

Pigs in the experimental and control groups presented similar average daily gains, daily feed intakes, feed conversion ratios, carcass weights, and intramuscular fat contents. There were no significant differences between the *CYP2E1* genotypes or nutritional or growth traits (Tables S1 and S2).

## 4 Discussion

CYP2E1 is a key enzyme in skatole metabolism in the pig liver. Recent cross‐species analysis confirmed that CYP2E1 exhibits a consistent pericentral zonation pattern in porcine liver, extending from zone 3 into the midzone (zone 2), with expression levels comparable to those observed in rodents and humans (Albadry et al. 2024). Its activity is affected by mutations (SNPs) in its coding gene (Lin et al. 2006; Moe et al. 2009; Mörlein et al. 2012; Rowe et al. 2014; Zadinová et al. 2017). To date, most studies have investigated the effects of SNPs on the levels of skatole and indole in boar adipose tissue or blood plasma, but not in feces. The level of skatole in feces could be an important indicator of metabolic and digestive processes. In addition, skatole from a soiling environment may be absorbed through the skin. Aluwé et al. (2011) described higher levels of skatole in adipose tissue from dirty boars than in that from clean boars.

The primary limitation of this study is the small sample size (*n* = 25), which resulted in limited statistical power and precluded testing of Diet × Genotype interaction effects. The modest number of animals reflects the pilot nature of the study—its principal aim was to determine whether a measurable relationship exists between fecal and adipose skatole, and whether fecal measurements could serve as a noninvasive detection tool in live boars. A further constraint was the methodological complexity of fecal sampling: samples had to be collected immediately after defecation to prevent skatole volatilization and cross‐contamination, which is not practicable with larger groups housed in a single pen. We acknowledge that these constraints limit the generalizability of our results, and that the findings should therefore be considered preliminary evidence requiring replication in larger, adequately powered trials.

### 4.1 Effects of Jerusalem Artichoke Feeding on the Levels of Skatole and Indole in Back Fat and Feces

The significant effects of the Jerusalem artichoke concentration in the diet on the skatole and indole concentrations in the adipose tissue, which are described in Table 2, are in accordance with previous studies (Vhile et al. 2012; Okrouhlá et al. 2020; Zadinová et al. 2021), confirming the decrease in skatole levels in boars fed with Jerusalem artichoke. According to Kozlowska et al. (2016), inulin positively influences the populations of *Lactobacillus* and *Bifidobacterium* in the large intestine, which has a positive effect on the decrease in skatole levels. Consistent with these findings, Lee et al. (2024) recently reported that dietary supplementation with 3% inulin numerically reduced skatole and indole concentrations in the adipose tissue of entire male pigs, further supporting the role of prebiotic fructans in modulating boar taint compounds.

Although there was a difference in the skatole content in feces between the control and experimental groups, this difference was not statistically significant. Based on the results of previous studies as well as the results of this study, the effect of Jerusalem artichoke becomes apparent only after a certain amount of time. A similar duration of feeding, which was sufficient to reduce the level of skatole in adipose tissue, was also reported by Vhile et al. (2012); Salmon and Edwards (2015) and Okrouhlá et al. (2020). Considering that the levels of skatole could differ across various boars, there may be boars in the herd that excrete large amounts of skatole. Aluwé et al. (2011) and Hansen et al. (1994) reported that pigs kept at high stocking density and in dirty pens presented higher skatole concentrations than pigs kept at low stocking density and in clean pens. They suggested that skatole may be absorbed through the skin and eventually accumulate in fat. For this reason, boars with high levels of skatole could affect animals with lower natural skatole levels. The solution could be to use selection on selected SNPs.

### 4.2 Effect of the *CYP2E1* SNP on Skatole and Indole Levels in Back Fat and Feces

A reduction in the accumulation of compounds by genetic selection in boars can be a valid strategy for preventing the surgical castration of male piglets (Faggion et al. 2023). The SNP of the *CYP2E1* gene c.1423A>G was included in this study. No significant effect on skatole levels in adipose tissue was confirmed for the SNPs studied. However, there was a significant trend that correlated with previous studies (Mörlein et al. (2012); Zadinová et al. (2017)).

An association with the level of skatole in feces was found for c.1423A>G polymorphism. The most favorable genotype, that is, the genotype with the lowest skatole level, was c.1423 *AA*. Conversely, in the case of genotype *GG*, the highest skatole level was recorded. These results could be due to the action of the *CYP* family genes in the small intestine or GIT. *CYP* expression has been described previously by Nielsen et al. (2017). The expression of *CYP1A* and *CYP3A* in the small intestine was confirmed. Although this study did not confirm *CYP2E1* expression, there could be a link between *CYP* family genes or some SNPs, which would explain the identified associations.

### 4.3 *CYP2E1* mRNA Expression in Boar Livers

The relationship between *CYP2E1* gene expression has been described in previous studies (Bee et al. 2020; Zadinová et al. 2021; Archer et al. 2024). The expression of *CYPs* in pigs is influenced by several factors, such as sex, breed, hormonal status, and the intake of specific feed components (Rasmussen and Zamaratskaia 2014). Notably, the effects of phytochemicals are concentration dependent (Burkina et al. 2018). Similarly, Marro et al. (2024) demonstrated that dietary supplementation with specific plant extracts (oregano, *Schisandra*, and garlic) modulated hepatic CYP enzyme activity, including CYP2E1, and significantly affected plasma and adipose skatole concentrations in entire male pigs, further supporting the concept that plant‐derived bioactive compounds can influence hepatic skatole metabolism. Rasmussen and Zamaratskaia (2014) described the beneficial effect of inulin enriched in chicory root on increasing the mRNA levels of *CYP2A19* and *CYP2E1* in boar primary hepatocytes. In addition to chicory, inulin is present in Jerusalem artichoke, which was used in this study. Moreover, in this study, the effect of inulin‐containing feed supplementation on the mRNA levels of *CYP2E1* was confirmed. Considering the equal representation of *CYP2E1* genotypes in the control and experimental groups, we can assume that the different mRNA expression levels are due primarily to external influences, that is, feeding. The effects of individual Jerusalem artichoke substances on the expression levels of *P450* family genes or enzymatic activity could be the subject of further study. In this case, it would be possible to label Jerusalem artichoke as a functional food that improves liver metabolism.

Another effect evaluated in this study that we hypothesized to influence *CYP2E1* mRNA expression is the genotype or the presence of specific SNPs. Archer et al. (2024) described the influence of several SNPs identified in the promoter region of the *CYP2E1* gene. A significant effect on *CYP2E1* mRNA expression, but not on CYP2E1 protein expression, was confirmed for two SNPs. The number of animals and the breed can influence the uncertainty of the results. The above mentioned study by Archer et al. (2024) evaluated three major swine breeds, Duroc, Landrace, and Yorkshire, in contrast to our study, which involved crossbreeds.

### 4.4 Growth Performance

Understanding the genetic relationships between skatole and indole concentrations and between carcass and meat quality is necessary to assess the potential effects of selecting against boar taint on other traits of interest (Faggion et al. 2023). The effects of the addition of the *CYP2E1* genotype and Jerusalem artichoke to boar diets on growth performance were not detected in this study. These results are consistent with those of previous studies (Vhile et al. 2012; Salmon and Edwards 2015; Aluwé et al. 2017; Zadinová et al. 2017). Because of the short feeding period of Jerusalem artichoke (14 days before slaughter), we did not expect any effect on growth parameters. This can be seen as a positive effect, as it is possible to use Jerusalem artichoke as a feed supplement.

## 5 Conclusion

In conclusion, the results of this study show that dietary supplementation with Jerusalem artichoke modulates hepatic metabolism by upregulating *CYP2E1* mRNA expression, which in turn contributes to a significant reduction of skatole levels in the adipose tissue of crossbred pigs. While previous research has established the impact of Jerusalem artichoke on gut microbiota composition, our findings provide novel evidence confirming its influence on liver metabolic pathways. Notably, skatole concentrations in pig feces were not significantly altered by Jerusalem artichoke supplementation; however, a pronounced effect of *CYP2E1* polymorphism on fecal skatole levels was observed. These results suggest that the *CYP2E1* c.1423G>A polymorphism contributes to individual variation in fecal skatole levels, indicating that genetic factors may play a role alongside dietary and environmental influences. However, as only one SNP was examined in this study, broader conclusions regarding the predominant role of genetic factors cannot be drawn and warrant further investigation into other polymorphisms within the *CYP* gene family expressed in the GIT. Future research should focus on elucidating the detailed mechanisms of skatole metabolism, the functional consequences of *CYP2E1* polymorphisms, and the expression profiles of other relevant *CYP* genes within the digestive system to better understand their roles in boar taint mitigation.

## Author Contributions

**Michal Šprysl:** resources, writing – review and editing. **Roman Stupka:** conceptualization, funding acquisition, resources, writing – review and editing. **Kateřina Zadinová:** conceptualization, formal analysis, investigation, methodology, project administration, resources, validation, visualization, writing – review and editing, writing – original draft. **Monika Okrouhlá:** formal analysis, investigation, validation, writing – review and editing. **Jan Calta:** data curation, formal analysis, investigation, validation, writing – review and editing, writing – original draft. **Jaroslav Čítek:** conceptualization, data curation, project administration, resources, software, validation, writing – review and editing. **Kateřina Urbancová:** formal analysis, investigation, writing – review and editing. **Antonín Stratil:** conceptualization, methodology, resources, supervision, writing – review and editing, writing – original draft. **Ivan Bahelka:** formal analysis, resources, visualization, writing – review and editing.

## Funding

This work was supported by the Ministry of Education, Youth and Sports of the Czech Republic, MSM 6046070901. The Ministry of Agriculture of the Czech Republic, QL25020030. Czech University of Life Sciences Prague, SV26‐5‐21320, SVJ24‐14‐21360. Institute of Animal Physiology and Genetics, AS CR, RVO 67985904.

## Ethics Statement

All experimental procedures were approved by the Ethics Committee of the Central Commission for Animal Welfare at the Ministry of Agriculture of the Czech Republic (Prague, Czech Republic) and were carried out in accordance with Directive 2010/63/EU for animal experiments and the Local Ethics Commission, case number 03/2017. The experiment was conducted at a testing station (11760061). All pigs were slaughtered according to the protocols for certified Czech slaughterhouses under the supervision of an independent veterinarian.

## Conflicts of Interest

The authors declare no conflicts of interest.

## Supporting information


**Table S1:** Effects of Jerusalem artichoke feeding on growth performance.
**Table S2:** Effects of CYP2E1 (c.1423G > A) genotypes on growth performance.

## Data Availability

Primers for genotyping of SNP NM_214421.1: c.1423G>A and for mRNA expression were taken from previously published studies (Zadinová et al., 2017; Zadinová et al., 2021 and Erkens et al., 2006). The data that support the findings of this study are available on request from the corresponding author. The data are not publicly available due to restrictions imposed by the funding bodies.
