# Supplementary material for: Effects of CYP2E1 Polymorphism and Dietary Supplementation With Jerusalem Artichoke on Skatole and Indole Levels in the Back Fat and Feces of Boars, and Liver mRNA Expression
Source: Food Sci Nutr. 2026 Sep 3;14(9):e72272. doi: 10.1002/fsn3.72272 (PMC13542426; doi:10.1002/fsn3.72272)
Supplement: Supplementary file 1 [file FSN3-14-e72272-s001.docx]

**Supplementary material**

**Supplementary Table S1:** Effects of Jerusalem artichoke feeding on growth performance

|  | Diet Group | | |
| --- | --- | --- | --- |
| Traits | **Control**  (n=12)  **LSM^1^ ± SE^2^** | **Experiment**  (n=13)  **LSM^1^ ± SE^2^** | ***p* value** |
| Live weight (kg) | 108.718 ± 9.51 | 108.346 ± 6.59 | NS |
| Daily feed intake (g) | 1688.886 ± 75.39 | 1659.022 ± 53.18 | NS |
| Feed conversion ratio (kg/kg) | 2.635 ± 0.85 | 2.265 ± 0.38 | NS |
| Average daily gain (g) | 805.291 ± 72.62 | 803.479 ± 49.48 | NS |
| Carcass weight (kg) | 85.983 ± 7.59 | 85.654 ± 5.72 | NS |
| Intramuscular fat (%) | 1.634 ± 0.29 | 1.594 ± 0.42 | NS |

^1^ LSM – least squares means

^2^ SE – standard error

**Supplementary Table S2:** Effects of *CYP2E1* (c.1423 G>A) genotypes on growth performance

|  | *CYP2E1* c.1423G>A | | | |
| --- | --- | --- | --- | --- |
| Traits | ***AA*** (n=5)  **LSM^1^ ± SE^2^** | ***AG*** (n=12)  **LSM^1^ ± SE^2^** | ***GG*** (n=8)  **LSM^1^ ± SE^2^** | **p value** |
| Live weight (kg) | 108.1 ± 7.96 | 109.842 ± 7.61 | 106.813 ± 9.12 | NS |
| Daily feed intake (g) | 1652.874 ± 81.27 | 1681.674 ± 59.30 | 1673.682 ± 69.68 | NS |
| Feed conversion ratio (kg/kg) | 2.755 ± 0.64 | 2.316 ± 0.65 | 2.437 ± 0.70 | NS |
| Average daily gain (g) | 801.587 ± 71.70 | 814.947 ± 56.21 | 790.178 ± 64.46 | NS |
| Carcass weight (kg) | 85.790 ± 6.65 | 87.000 ± 6.78 | 84.050 ± 6.58 | NS |
| Intramuscular fat (%) | 1.737 ± 0.48 | 1.702 ± 0.37 | 1.402 ± 0.14 | NS |

^1^ LSM – least squares means

^2^ SE – standard error
